# Functional Band and Loop Space Maintainers in Children

**DOI:** 10.1155/2019/4312049

**Published:** 2019-04-24

**Authors:** V. Vinothini, A. Sanguida, A. Selvabalaji, G. S. Prathima, M. Kavitha

**Affiliations:** ^1^Vinu Dental Clinic, Erode 638 452, Gobichettipalayam, India; ^2^Department of Paedodontics and Preventive Dentistry, Indira Gandhi Institute of Dental Sciences, Sri Balaji Vidyapeeth, Pillaiyarkuppam, Puducherry 607 402, India

## Abstract

Premature loss of teeth in children leads to space loss and affects arch integrity. The band and loop space maintainer is used in majority of patients requiring single tooth space maintenance in both primary and mixed dentitions. It preserves the proximal dimensions, but it is nonfunctional. This paper describes a method to modify the conventional band and loop space maintainer into a functional one and reports its clinical application and follow-up in five children.

## 1. Introduction

A healthy primary dentition preserves space for permanent teeth and maintains arch integrity. Diverse dietary patterns make children more susceptible to dental caries and result in premature loss of primary teeth, thereby necessitating placement of space maintainers [1, 2]. Space maintainers guide the eruption of the permanent teeth and obviate the need for complex orthodontic treatment later. The most commonly used fixed space maintainer is the band and loop space maintainer. Although it is easier to construct, economical, and consumes less chair-side time, it fails to restore the occlusal function of the lost tooth. Hence, an attempt is made to modify the appliance so as to make it functional.

## 2. Technique of Fabrication of the Functional Band and Loop Space Maintainer

**Figure 1:**
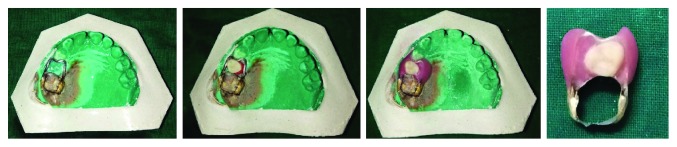
Steps in the construction of a functional band and loop space maintainer.

The first step is to construct a conventional band and loop space maintainer in the region of premature tooth loss. This is followed by the placement of an acrylic tooth in the edentulous area of the cast and stabilization with modeling wax. The occlusion is then checked with the cast of the opposing arch and adjusted. Cold cure acrylic is used to attach the pontic to the loop. The completed appliance is then finished and polished (Figure 1). Trial fit is done in patient's mouth, and the appliance is checked for the presence of soft tissue irritation or occlusal interferences and adjusted accordingly. The final cementation of the appliance is done.

## 3. Case 1

**Figure 2:**
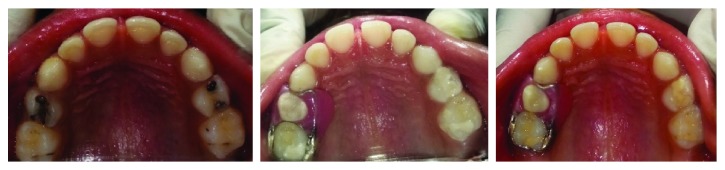
Preoperative, immediate postoperative, and one-year postoperative occlusal views of the maxillary arch with the functional band and loop space maintainer in the tooth 54 region.

A 6-year-old female patient reported to the Department of Pedodontics with a chief complaint of pain in the upper right back tooth region. The right maxillary deciduous first molar was carious with the resorption of more than 2/3^rd^ of its roots and hence had to be extracted. Model analysis was done followed by the placement of a fixed functional band and loop space maintainer (Figure 2).

## 4. Case 2

**Figure 3:**
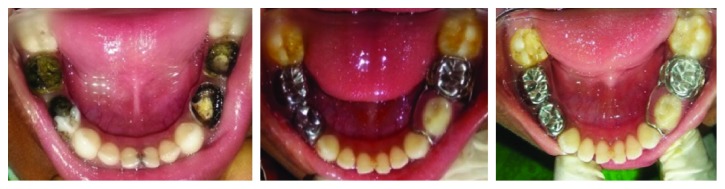
Preoperative, immediate postoperative, and one-year postoperative occlusal views of the mandibular arch with the functional band and loop space maintainer in the tooth 74 region.

A 6-year-old boy had grossly decayed teeth 74, 75, 84, and 85. Radiograph of 74 showed poor prognosis and was extracted. Pulpectomy was done for the remaining teeth, and teeth were restored with stainless steel crowns. A functional band and loop space maintainer was cemented in relation to 74. Patient was recalled after three months, and it was found that there was no soft tissue irritation or dislodgement of the appliance (Figure 3).

## 5. Case 3

**Figure 4:**
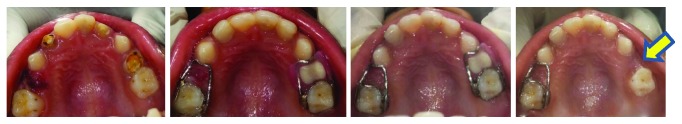
Preoperative, immediate postoperative, and one-year postoperative occlusal views of the maxillary arch with the conventional band and loop space maintainer in the tooth 54 region and functional band and loop space maintainer in the tooth 64 region. Note the absence of tissue changes (arrow).

A 7-year-old female patient reported with the chief complaint of multiple decayed teeth and also with a history of extraction of the decayed right upper back tooth. Clinical examination revealed grossly decayed 53 and 64 and clinically missing 54. Radiograph of 64 revealed a poor prognosis and hence was extracted. Following model analysis, a conventional band and loop space maintainer in the 54 region and a functional band and loop space maintainer in the 64 region were cemented. Tooth 53 was endodontically treated and esthetically restored with composite resin (Figure 4). Patient was reviewed every three months, and she reported that the fixed functional band and loop space maintainer helped her to chew comfortably. There was no mucosal irritation in relation to the appliance.

## 6. Case 4

**Figure 5:**
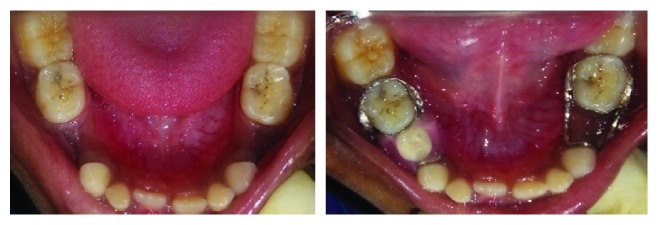
Preoperative and immediate postoperative views of the mandibular arch with the conventional band and loop space maintainer in the tooth 74 region and functional band and loop space maintainer in the tooth 84 region.

A 6-year-old female patient reported with clinically missing 74 and 84. Past dental records revealed the extraction of teeth 74 and 84 due to caries four months and two months earlier, respectively. History of difficulty in mastication was also reported. Model analysis was done. There was no space loss; hence, it was planned to maintain space with the functional band and loop space maintainer in relation to 84 and conventional one in relation to 74. Patient was recalled every three months for review, and she felt comfortable to chew on the right side (Figure 5).

## 7. Case 5

**Figure 6:**
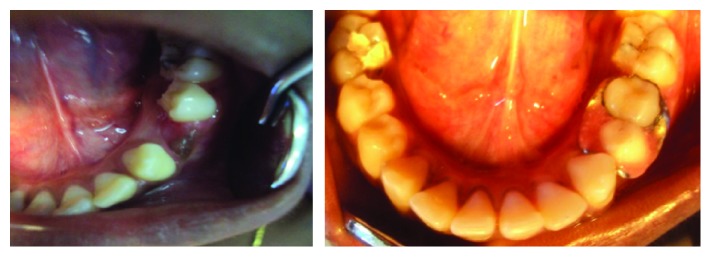
Preoperative and immediate postoperative occlusal views of the mandibular arch with the functional band and loop space maintainer in the tooth 34 region.

A 13-year-old girl reported to the department with complaint of several broken teeth and one lost permanent tooth. Parents gave history of trauma to the chin region due to accidental fall from a tractor ten days before reporting. Her medical history was not remarkable. On intraoral examination, tooth numbers 15, 16, 25, 26, 35, 36, and 45 had sustained uncomplicated crown fractures. Tooth number 34 was clinically missing, and tooth number 46 showed a complicated crown fracture. Panoramic radiograph confirmed the avulsion of 34 and showed no evidence of fracture involving maxilla or mandible. It was planned to restore tooth numbers 15, 25, 35, and 45 with composite restorations, perform RCT in 46, and restore 16, 26, 36, and 46 with stainless steel crowns as they had extensive tooth structure loss due to the impact of trauma. A functional space maintainer (band and loop with acrylic pontic of tooth number 34) was planned as an interim prosthesis and a space maintainer in the tooth 34 region. As the tooth 35 had extensive tooth loss on lingual aspect, the band would also help in the retention of the composite restoration till future definitive restorative management (Figure 6).

## 8. Discussion

Band and loop appliance is a versatile space maintainer for maintaining space due to premature loss of a single tooth [3]. It has shown good success rates [4–6]. However, the disadvantage is that it is not functional. Apart from maintaining the mesiodistal dimension of the space created by premature loss of a tooth, a space maintainer should aid in mastication and prevent overeruption of the opposing tooth or teeth. It should also be simple, not interfere with normal occlusal adjustments, or restrict normal growth and development [2]. However, no space maintainer fulfills all the ideal requirements. A new technique of the fabrication of a functional band and loop space maintainer was described in this report.  Table 1 lists the pros and cons of the modified space maintainer compared to the traditional band and loop space maintainer.

In order to compare the acceptability of the modified band and loop space maintainer with that of the conventional one, two children were given both types. The two children reported satisfaction with the modified band and loop space maintainer and found that it is comfortable for eating.

All the children were recalled every three months. This appliance design did not interfere with oral hygiene maintenance nor did it causes soft tissue irritation, discomfort, or food lodgement. There were no reports of fracture of the appliance. However, it should be noted that the maximum follow-up period was only one year; hence, long-term follow-up is essential to evaluate the longevity of the appliance.

## 9. Conclusion

Managing space and at the same time improving the masticatory function and maintaining arch integrity in early loss of the primary teeth are challenging tasks. The functional band and loop space maintainer described in this report will be a good choice for use in premature loss of a single tooth in very young children.

## Figures and Tables

**Table 1:** 

Advantages
(1) Aids in mastication. (2) Prevents supraeruption of the opposing tooth. (3) Distribution of occlusal forces on the pontic and hence less chance of loop distortion/slippage and impingement in gingiva. (4) Prevents the development of abnormal tongue habits.
Limitations and possible solutions
(1) Direct visualization of the eruption of the successor is not possible. (a) Hence, long-term and frequent follow-ups (preferably using radiographs—RVG) are required. Periodic removal and clinical inspection for signs of the eruption of the successor or tissue irritation followed by recementation should be performed as would be done for any traditional space maintainer. (b) Parents should be informed that the pontic alone will be removed from the appliance when the child approaches the eruption age of the successor or if there is clinical or radiographic evidence of impending tooth emergence. (2) Cement loss and solder failure can be possible reasons for failure of this appliance. Hence, quality designing of the appliance, close supervision, and frequent follow-ups at 2-4 month interval are imperative.
